# Association between Dietary Patterns and Cardiometabolic Multimorbidity among Chinese Rural Older Adults

**DOI:** 10.3390/nu16172830

**Published:** 2024-08-24

**Authors:** Fangfang Hu, Wenzhe Qin, Lingzhong Xu

**Affiliations:** 1 Center for Health Management and Policy Research, School of Public Health, Cheeloo College of Medicine, Shandong University, Jinan 250012, China; hufang@mail.sdu.edu.cn; 2 National Health Commission (NHC) Key Laboratory of Health Economics and Policy Research, Shandong University, Jinan 250012, China; 3 Center for Health Economics Experiment and Public Policy Research, Shandong University, Jinan 250012, China

**Keywords:** dietary patterns, cardiometabolic multimorbidity, rural older adults, prevalence, latent profile analysis

## Abstract

Background: The global population is aging rapidly, leading to an increase in the prevalence of cardiometabolic multimorbidity (CMM). This study aims to investigate the association between dietary patterns and CMM among Chinese rural older adults. Methods: The sample was selected using a multi-stage cluster random sampling method and a total of 3331 rural older adults were ultimately included. Multivariate logistic regression analysis was used to examine the association between the latent dietary patterns and CMM. Results: The prevalence of CMM among rural older adults was 44.64%. This study identified four potential categories: “Low Consumption of All Foods Dietary Pattern (C1)”, “High Dairy, Egg, and Red Meat Consumption, Low Vegetable and High-Salt Consumption Dietary Pattern (C2)”, “High Egg, Vegetable, and Grain Consumption, Low Dairy and White Meat Consumption Dietary Pattern (C3)” and “High Meat and Fish Consumption, Low Dairy and High-Salt Consumption Dietary Pattern (C4)”. Individuals with a C3 dietary pattern (OR, 0.80; 95% CI, 0.66–0.98; *p* = 0.028) and a C4 dietary pattern (OR, 0.70; 95% CI, 0.51–0.97; *p* = 0.034) significantly reduced the prevalence of CMM compared with the C1 dietary pattern. Conclusions: Rural older adults have diverse dietary patterns, and healthy dietary patterns may reduce the risk of CMM.

## 1. Introduction

As the global population ages at an accelerated pace and life expectancy extends, the prevalence of multimorbidity has become increasingly common [1]. The elderly population commonly faces the coexistence of two or more cardiometabolic diseases simultaneously, which is called cardiometabolic multimorbidity (CMM) [2,3]. CMM encompasses a range of conditions including hypertension, type 2 diabetes, dyslipidemia, cerebral infarction, stroke, ischemic heart disease, and other cardiovascular disease [4,5], which collectively heighten the risk of cardiovascular diseases and mortality [6]. CMM has emerged as a widespread and stable pattern of multimorbidity, with its prevalence sharply on the rise worldwide [7]. The challenges posed by CMM have been widely acknowledged as one of the significant issues in the public health domain [8]. A study indicated that individuals with CMM face double the mortality risk compared to those without such comorbidities [9]. CMM impacts not only the rehabilitation process of patients but may also exacerbate disease prognosis, increase the medical burden, and severely diminish the quality of life [10,11]. However, the prevalence of CMM in rural populations remains insufficiently explored. Compared to urban areas, rural areas often exhibit differences in lifestyle factors, accessibility to healthcare, and socioeconomic conditions, which may influence the prevalence of CMM [12,13,14]. Therefore, investigating the prevalence of CMM among rural older adults is crucial for the prevention and management of CMM.

Dietary patterns, as multidimensional reflections of diet, encompass the quantity and proportion of various foods in the diet, rather than focusing on a single nutrient or food item [15]. Recognized as key determinants of cardiometabolic health, dietary patterns are closely associated with the risk of various diseases, including metabolic syndrome, cardiovascular diseases, stroke, type 2 diabetes, and other chronic metabolic disorders [16]. The available studies predominantly focus on analyzing individual dietary behaviors, such as the consumption of vegetables, fruits, and meats, and their impact on health outcomes [17,18,19]. Few studies have explored the dietary patterns among older adults [20,21]. While several studies have identified various dietary patterns associated with cardiometabolic risk [22], knowledge about the dietary patterns and their health impacts among rural elderly populations remains limited. Rural populations often experience unique dietary patterns influenced by local agricultural practices, economic constraints, and cultural habits. A study suggested that close adherence to a Mediterranean diet seems to play a key role in age-related disease prevention and in attaining longevity among rural population [23]. These dietary patterns, in turn, play an essential role in shaping health outcomes [24]. Despite the acknowledged importance of diet in the prevention and management of cardiometabolic diseases, comprehensive research on the specific dietary patterns of rural elderly individuals and their association with cardiometabolic health is still lacking. In particular, a large body of research indicated that dietary patterns increase the risk of individual cardiometabolic diseases [25]. However, the extent to which dietary patterns contribute to CMM, especially in rural older adults, remains unclear. Clinical trials often exclude patients with multimorbidity, and observational studies typically focus on single disease outcomes, thus limiting our understanding of CMM risk factors [26]. Therefore, understanding the dietary patterns of rural older adults and exploring their association with CMM is essential for developing targeted nutritional interventions and improving health outcomes.

To fill these research gaps, we conducted a comprehensive analysis of a representative sample of rural older adults in China. This study aimed to: (1) reveal the prevalence of CMM among rural older adults; (2) identify the latent dietary patterns of rural older adults; and (3) investigate the association between dietary patterns and CMM. The findings are expected to provide in-depth insights into the specific cardiometabolic health challenges faced by rural older adults and guide future dietary interventions aimed at reducing the burden of CMM.

## 2. Materials and Methods

### 2.1. Study Design and Sample

The sample was selected using a multi-stage cluster random sampling method. First, based on the level of socioeconomic development (measured by per capita gross domestic product, GDP) and geographical location, one prefecture-level city was selected from each of the eastern (Weifang City), central (Tai’an City), and western (Dezhou City) regions of Shandong Province. Next, in each prefecture-level city, one county-level city was randomly selected: Gaomi City in the east, Xintai City in the center, and Laoling City in the west. Then, proportional probability sampling was used to select three to four townships or sub-districts from each county-level city, including four townships/sub-districts from Gaomi City, four from Xintai City, and three from Laoling City, totaling 11 townships/subdistricts. Subsequently, six villages were randomly selected from each township or sub-district. Finally, 40 households were randomly selected from each sample village. In the end, the survey aimed to investigate 2640 households and actually investigated 2682 households, with 2664 valid questionnaires collected. The inclusion criteria were rural elderly residents over 60 years old who are conscious and able to communicate clearly. The exclusion criteria were elderly individuals with a history of mental illness or those diagnosed with dementia. In this study, a total of 3332 rural older adults aged 60 and above participated in the survey. After excluding missing values, 3331 rural older adults were included in this study.

### 2.2. Assessment of CMM

Cardiometabolic diseases are a group of disorders that include cardiovascular, renal, metabolic, thrombosis, and inflammation abnormalities. The typical features of these diseases are insulin resistance, impaired glucose tolerance, dyslipidemia, and hypertension [4]. This study primarily focuses on comorbidities, such as hypertension, type 2 diabetes, dyslipidemia, cerebral infarction, stroke, ischemic heart disease, and other cardiovascular diseases. The presence of CMM was determined through self-reported medical histories obtained via face-to-face questionnaires with researchers. Participants were categorized as either having no CMM or having CMM (defined as having two or more types of comorbidities).

### 2.3. Dietary Assessment

The dietary intake of rural older adults was assessed by asking participants about the frequency of their consumption of the following foods in the past 12 months: (1) red meat; (2) white meat; (3) fish and seafood; (4) fresh vegetables; (5) fresh fruits; (6) eggs; (7) legumes and their products; (8) dairy and its products; (9) grains; (10) high-sugar foods; (11) fried and deep-fried foods; (12) high-salt foods. Each type of food was categorized into 8 frequency groups, ranging from low to high: never, less than once a month, 1–3 times a month, once a week, 2–3 times a week, 4–6 times a week, 1–2 times a day, and 3 or more times a day [27]. Older adults selected one of the 8 frequency categories that best fits their situation by recalling their intake of each food in the past 12 months, in order to determine their average intake frequency of various foods.

### 2.4. Covariates

Covariates included age (continuous variable); sex (male, female); highest education (illiteracy, primary school, middle school, high school and higher); marital status (with a spouse, without a spouse); living arrangement (living alone, living only with spouse, living with spouse and children); personal income (<5000 (Q1), 5000~10,000 (Q2), ≥10,000 (Q3)); smoking status (smoker, non-smoker); drinking status (drinker, non-drinker); present occupation (no, farming, non-farming occupations); medical insurance type (Basic Medical Insurance for Urban Employee (UEBMI), Basic Medical Insurance for Urban and Rural Residents (RBMI), Others); online frequency (Never, 1~4 times per month, Every day); obesity status (BMI < 18.5 kg/m^2^ (underweight), 18.5 ≤ BMI ≤ 23.9 kg/m^2^ (normal), 24 ≤ BMI ≤ 27.9 kg/m^2^ (overweight), BMI ≥ 28 kg/m^2^ (obesity)); amount of exercise (little, middle, big), following a previous study methodology.

### 2.5. Statistical Analysis

A database was established using EpiData 3.1 software. Statistical analysis was performed using IBM SPSS 25.0 and Mplus version 8.1 software. The categorical variables were described as amount with percentage, and the chi-square test was used to compare the prevalence of CMM among rural older adults with different demographic characteristics. The continuous variables were described as mean and standard deviation, and T-tests were used to compare demographic differences. Latent profile analysis (LPA) was employed to identify dietary patterns [28]: (1) Firstly, the consumption frequency of 12 types of food (as continuous variables) were standardized using Z-scores. LPA was conducted to establish five latent classes from few to many; (2) Model fit indices Akaike information criterion (AIC), Bayesian information criterion (BIC), adjusted Bayesian information criterion (aBIC)), classification accuracy indices (entropy), and model evaluation indices (Lo-Mendell-Rubin adjusted likelihood ratio test (LMRT), bootstrapped likelihood ratio test (BLRT)) were used to comprehensively evaluate and select the best model based on interpretability. (3) Generally, smaller values of AIC, BIC, and aBIC indicate a better model fit, and higher entropy indicates better classification accuracy (the minimum standard is 0.8). (4) Statistically significant LMR and BLRT values indicate that model K is better than model K-1. Additionally, the final model selection should consider model simplicity, interpretability, and theoretical significance, and ensure that the number of individuals in each class is not less than 5% of the total sample. Multivariate logistic regression analysis was used to examine the association between dietary patterns and CMM among rural older adults. Two models were established with CMM (0 = no, 1 = yes) as the dependent variable and latent classes of dietary patterns as the independent variable (with latent class 1 as the reference group). Model 1 did not control for confounding variables, and Model 2 controlled for demographic variables affecting CMM among rural older adults. We set the statistical significance level at *p* < 0.05.

## 3. Results

### 3.1. Characteristics of Study Participants

Table 1 shows the prevalence of CMM and the characteristics of the study participants. The prevalence of CMM among rural older adults was 44.64%. A total of 3331 older adults aged 60 and above (41.67% males and 58.33% females) were included in this study. The average age of all participants was (70.89 ± 5.88) years old. In addition to live arrangement, medical insurance type, online frequency, and amount of exercise, the prevalence of CMM was significantly different according to age, sex, highest education, marital status, personal income, smoking status, drinking status, present occupation, and obesity status.

### 3.2. Latent Profile Analysis Dietary Patterns

The results of the latent profile analysis are shown in Table 2. AIC and BIC decreased as the number of profiles increased, indicating a better fit for the models with more profiles. A higher entropy also indicates a stronger model fit. Entropy was highest for the 3-profile solution (0.90). The entropy was >0.88 for each other solution, except for the 2-profile solution. The *p* values for the LMRT indicated that a 5-profile solution fit as well as a 4-profile solution; however, one of the profiles in the 5-profile solution contained less than 5% of the sample. Based on these criteria, a 4-profile solution was considered optimal (Entropy = 0.88, BIC = 108527.24, LMRT < 0.001, BLRT < 0.001).

Additionally, the average probabilities of the participants being assigned to the four latent classes are presented in Table 3. For each rural older adult, the average probability of being assigned to their respective class was above 0.87, while the probability of being assigned to any other class was below 0.13. This further supports the accuracy of the four-class model.

**Figure 1:**
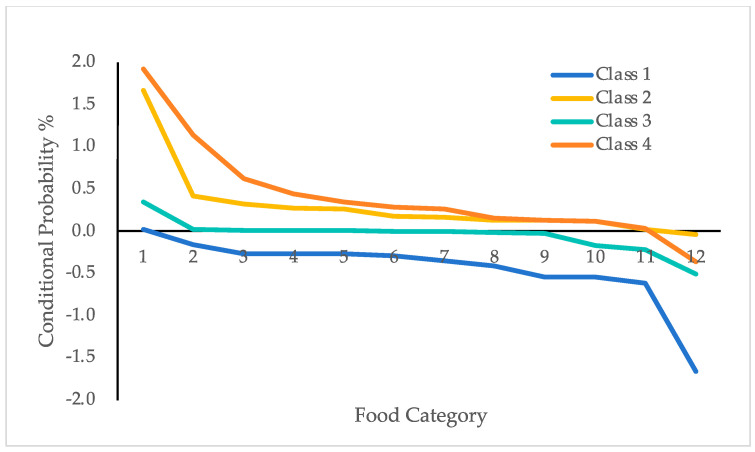
Distribution of the estimated conditional mean of 4 latent classes.

The distribution of scores of dietary patterns of latent classes across 12 food categories were shown in Figure 1. Items of the same dimension were grouped together, and the four classes were named based on their score characteristics for each food category. Class 1 had relatively low scores across all food categories, indicating that these rural older adults had a relatively low intake of all food types. Therefore, this class was named “Low Consumption of All Foods Dietary Pattern,” with a total of 571 individuals, accounting for 17% of the sample. Class 2 had relatively high scores for dairy, eggs, and red meat, and relatively low scores for vegetables and high-salt foods, indicating that these rural older adults had a higher intake of dairy, eggs, and red meat, and a lower intake of vegetables and high-salt foods. This class was named “High Dairy, Egg, and Red Meat Consumption, Low Vegetable and High-Salt Consumption Dietary Pattern,” with a total of 754 individuals, accounting for 23% of the sample. Class 3 had relatively high scores for eggs, vegetables, and grains, and relatively low scores for dairy and white meat, indicating that these rural older adults had a higher intake of eggs, vegetables, and grains, and a lower intake of dairy and white meat. This class was named “High Egg, Vegetable, and Grain Consumption, Low Dairy and White Meat Consumption Dietary Pattern,” with a total of 1770 individuals, accounting for 53% of the sample. Class 4 had relatively high scores for white meat, red meat, and fish, and relatively low scores for dairy and high-salt foods, indicating that these rural older adults had a higher intake of meat and fish, and a lower intake of dairy and high-salt foods. This class was named “High Meat and Fish Consumption, Low Dairy and High-Salt Consumption Dietary Pattern,” with a total of 236 individuals, accounting for 7% of the sample.

### 3.3. Prevalence of CMM by Dietary Patterns

The results of the prevalence of CMM across different dietary patterns were shown in Table 4. The differences in the prevalence of CMM among rural older adults across different dietary patterns were statistically significant (*p* < 0.05). The prevalence of CMM in Class 4 (39.83%) was lower than in Class 1 (48.34%), Class 2 (46.82%), and Class 3 (43.16%).

### 3.4. Association between Dietary Patterns and the Prevalence of CMM among Rarul Older Adults

The association between dietary patterns and the prevalence of CMM are presented in Table 5. The analysis was performed using the same adjustment models used in the multivariate logistic regression analysis. In Model 1, individuals with a C3 dietary pattern (OR, 0.81; 95% CI, 0.67–0.98; *p* = 0.031) and a C4 dietary pattern (OR, 0.71; 95% CI, 0.52–0.96; *p* = 0.028) were significantly less likelihood of having CMM as compared to those with C1 dietary patterns. In Model 2, individuals with a C3 dietary pattern (OR, 0.80; 95% CI, 0.66–0.98; *p* = 0.028) and a C4 dietary pattern (OR, 0.70; 95% CI, 0.51–0.97; *p* = 0.034) were significantly less likelihood of having CMM as compared to those with a C1 dietary pattern.

## 4. Discussion

In this study, the prevalence of CMM was 44.64% among rural older adults in Shandong Province, China. This rate is higher than the prevalence of CMM reported in other studies. Jin et al. reported that the prevalence of CMM in a c pooled multi-cohort study was 8.8% [29], which is a mere 20% of the rate found in our study. The China Health and Retirement Longitudinal Study (CHARLS) found that among 7125 participants, 239 incident cases of CMM were identified during a median follow-up of 7.0 years [30]. A meta-analysis indicated that the prevalence of CMM in middle-aged and older adults was 14.6% [31]. The cross-sectional study determined that the overall crude prevalence of CMM was 11.5% among 101,973 Chinese adults [32]. The prevalence of CMM was 24.5% among middle-aged and older adults [5]. Guo et al. reported that the prevalence of CMM among older adults aged 60 and above in China is 33.94% [33]. Another study indicated that the prevalence of CMM was 33.1% among the total sample of 4832 Chinese women [34]. This study focuses on rural older adults, where poor health awareness and unhealthy lifestyles have led to a higher prevalence of CMM. This body of evidence suggests that China is experiencing an epidemic of cardiometabolic comorbidities, which may be related to demographic shifts, economic development, and changes in lifestyle. Additionally, the prevalence of CMM is also high in the South Korea (50%) [35], Turkey (44%) [36], and the United States (35%) [37]. In contrast, a survey-based study in Canada found that only 3.5% of adults aged 50 and above had CMM [38]. A cohort analysis of the UK Biobank cohort showed that 4.7% of the follow-up population had CMM [14]. Within the Swedish twin registry, 4.7% individuals aged ≥ 60 had CMM [10]. These discrepancies might be explained by the variations in sample regions, inconsistencies in the definition of CMM, differences in inclusion criteria, and the application of different sampling methods [3].

This study identified four potential categories among rural older adults: “Low Consumption of All Foods Dietary Pattern”, “High Dairy, Egg, and Red Meat Consumption, Low Vegetable and High-Salt Consumption Dietary Pattern”, “High Egg, Vegetable, and Grain Consumption, Low Dairy and White Meat Consumption Dietary Pattern”, and “High Meat and Fish Consumption, Low Dairy and High-Salt Consumption Dietary Pattern”. Among these, the “High Egg, Vegetable, and Grain Consumption, Low Dairy and White Meat Consumption Dietary Pattern” had the highest number of rural older adults. This group’s diet was rich in eggs, vegetables, and grains, which was often a result of self-sufficiency in farming and animal husbandry, providing a reliable source of fresh, familiar produce. The practice of cultivating and rearing one’s food not only cuts down on grocery expenses but also holds significant cultural and traditional value in rural societies. It is perceived as a pathway to a healthier and more natural diet, free from the additives found in commercial food products. Conversely, the “High Meat and Fish Consumption, Low Dairy and High-Salt Consumption Dietary Pattern” had the fewest individuals, which is related to the living standards of rural older adults, who consume less meat and fish daily due to economic constraints. In a cohort study of Chinese older adults, Pei et al. used exploratory factor analysis to identify three dietary patterns: the vegetable–egg–beans–milk pattern, the meat–fish pattern, and the salt-preserved vegetable–garlic pattern [20], which are similar to the dietary patterns in this study. Chen et al. found that three dietary patterns (vegetable, meat, and traditional) were identified in Chinese older adults [21]. Similarly, another study classified food intake information into three categories: healthful plant foods, less-healthful plant foods, and animal foods, offering a comprehensive view of the dietary habits of the elderly population [39].

This study found that, compared with the “Low Consumption of All Foods Dietary Pattern”, both the “High Egg, Vegetable, and Grain Consumption, Low Dairy and White Meat Consumption Dietary Pattern” and “High Meat and Fish Consumption, Low Dairy and High-Salt Consumption Dietary Pattern” were associated with a lower risk of CMM. The “High Egg, Vegetable, and Grain Consumption, Low Dairy and White Meat Consumption Dietary Pattern” is rich in protein from eggs, vitamins and minerals from vegetables, and dietary fiber from grains, which helps reduce the risk of chronic diseases [40,41]. Previous studies have found that a high consumption of vegetables and whole grains is beneficial in preventing various cardiometabolic diseases, consistent with the results of this study [42,43,44]. Veldheer et al. found that for individuals over 65 years old, those who engage in gardening tend to have a higher intake of fresh vegetables, which helps prevent cardiovascular diseases and diabetes [45]. The Mediterranean diet, characterized by the daily intake of fruits, vegetables, nuts, beans, and unrefined grains, has been strongly and consistently confirmed by the previous studies to benefit cardiovascular health, including reducing the incidence of cardiovascular outcomes [46]. Eggs are a major source of dietary cholesterol, which plays important roles in bile acid formation, cell membrane composition, and hormone synthesis [47]. The “High Meat and Fish Consumption, Low Dairy and High-Salt Consumption Dietary Pattern” provides high-quality protein and omega-3 fatty acids, which may play a key role in improving health. Although dairy intake is low, the benefits of consuming nutrient-rich meat and fish may outweigh the negative impacts of low dairy intake, thereby exerting a protective effect against CMM. Previous research has shown that moderate consumption of meat and fish is associated with improved health outcomes, supporting our results [48]. A study focusing on older adults aged 70–79 found that a dietary pattern including low-fat dairy, fruits, whole grains, poultry, fish, and vegetables may be associated with higher insulin sensitivity, an effective method for preventing diabetes [49]. Among other animal protein sources, moderate fish consumption is also supported by the latest evidence, although there might be sustainability concerns [50]. The Japanese dietary pattern, characterized by high consumption of soy products, fish, seaweed, vegetables, fruits, and green tea, is associated with lower cardiovascular mortality [51]. Kim et al. also found that a high intake of fish and seafood can reduce the risk of metabolic syndrome [52]. Studies at home and abroad have pointed out that red meat is a risk factor for hypertension [53,54], but these studies did not control for other dietary factors, so their conclusions may actually be due to reduced white meat intake. In fact, many previous studies have confirmed that the DASH diet and the Mediterranean diet can reduce the risk of hypertension. The DASH diet emphasizes reducing animal fat intake, while the Mediterranean diet advocates for sufficient intake of fish and seafood [55]. Both diets favor white meat when choosing meat intake, which aligns with the conclusions of this study. There is strong evidence that a higher consumption of vegetables, fruits, whole grains, low-fat dairy, and seafood, and a lower consumption of red and processed meat, refined grains, and sugar-sweetened foods and beverages dietary patterns can reduce the risk of cardiovascular disease [56]. Furthermore, the “Low Consumption of All Foods Dietary Pattern” may represent individuals facing significant economic hardship, leading to insufficient dietary intake and malnutrition, thus increasing the risk of CMM. Compared to the “Low Consumption of All Foods Dietary Pattern”, the “High Egg, Vegetable, and Grain Consumption, Low Dairy and White Meat Consumption Dietary Pattern” and the “High Meat and Fish Consumption, Low Dairy and High-Salt Consumption Dietary Pattern” indicate relatively better economic conditions, allowing for a more diverse and nutritious diet. Previous research has pointed out the correlation between socioeconomic status and diet quality, supporting our findings [57]. Therefore, attention should be given to dietary diversity among older adults, advocating for increased consumption of vegetables and grains to supplement dietary fiber, enhanced protein intake, and more consumption of eggs, white meat, and fish to reduce the incidence of CMM.

The strengths of our study include the use of Latent Profile Analysis to categorize complex dietary data into several meaningful groups, thereby providing a better understanding of the different dietary patterns among rural older adults. This classification method can reveal subtle differences that traditional analyses might overlook. Additionally, by focusing on rural older adults, we address a population that has been underrepresented in previous research. The dietary habits and health status of rural older adults are unique, making this study of significant social and public health importance. Most importantly, this research fills a gap by analyzing the relationship between dietary patterns and the prevalence of CMM among rural older adults, providing essential empirical support for the development of public health interventions and health policies.

However, this study has several limitations. Firstly, the research conclusions are based on cross-sectional data, which cannot confirm the causal association between dietary patterns and CMM. Future research should use longitudinal study designs to analyze the impact mechanisms between dietary patterns and CMM among rural older adults. Secondly, sample selection may have selection bias. Our sample mainly comes from rural older adults in a specific area, so the external validity of the research results may be limited and difficult to generalize to other populations. Thirdly, this study relies on self-report from participants during the data collection process, which may result in recall bias. To enhance the accuracy and reliability of the research findings, future studies could consider combining self-reported data with objective biomarkers.

## 5. Conclusions

In conclusion, this study showed that the prevalence of CMM was high among rural older adults in Shandong Province, China. We classified dietary patterns into four categories: “Low Consumption of All Foods Dietary Pattern”, “High Dairy, Egg, and Red Meat Consumption, Low Vegetable and High-Salt Consumption Dietary Pattern”, “High Egg, Vegetable, and Grain Consumption, Low Dairy and White Meat Consumption Dietary Pattern”, and “High Meat and Fish Consumption, Low Dairy and High-Salt Consumption Dietary Pattern”. Among these, the “High Egg, Vegetable, and Grain Consumption, Low Dairy and White Meat Consumption Dietary Pattern” and “High Meat and Fish Consumption, Low Dairy and High-Salt Consumption Dietary Pattern” were significantly associated with a reduced risk of CMM. Our findings emphasized the importance of dietary diversity, particularly the intake of eggs, fresh vegetables, grains, white meat, and fish and seafood, in influencing cardiometabolic health. It is noteworthy that dietary patterns associated with reducing CMM risk are characterized by low dairy intake. However, the exact impact of dairy fat content on CMM development still needs to be fully understood, indicating the need for further research. It is also important to consider the role of low-fat dairy alternatives in future dietary guidelines to promote overall strategies for maintaining cardiometabolic health.

## Figures and Tables

**Table 1:** Characteristics of participants and prevalence of CMM.

Characteristics	Total (*n* = 3331)	CMM (*n* = 1487)	NO-CMM (*n* = 1844)	*t/χ*2	*p*
Age (years)	70.89 (5.88)	71.32 (5.69)	70.54 (6.01)	−3.85	<0.001
Sex				52.63	<0.001
Male	1388 (41.67%)	517 (37.25%)	871 (62.75%)		
Female	1943 (58.33%)	970 (49.92%)	973 (50.08%)		
Highest education				14.09	0.003
Illiteracy	1452 (43.59%)	691 (47.59%)	761 (52.41%)		
Primary School	1127 (33.83%)	491 (43.57%)	636 (56.43%)		
Middle school	565 (16.96%)	240 (42.48%)	325 (57.52%)		
High school and higher	187 (5.61%)	65 (34.76%)	122 (65.24%)		
Marital status				6.21	0.013
With a spouse	2539 (23.78%)	384 (48.48%)	408 (51.52%)		
Without a spouse	792 (76.22%)	1103 (43.44%)	1436 (56.56%)		
Live arrangement				5.26	0.072
Living alone	626 (18.79%)	302 (48.24%)	324 (51.76%)		
Living only with spouse	2205 (66.20%)	977 (44.31%)	1228 (55.69%)		
Living with spouse and children	500 (15.01%)	208 (41.60%)	292 (58.40%)		
Personal income				33.29	<0.001
Q1	1794 (53.86%)	880 (49.05%)	914 (50.95%)		
Q2	892 (26.78%)	368 (41.26%)	524 (58.74%)		
Q3	645 (19.36%)	239 (37.05%)	406 (62.95%)		
Smoking status				30.61	<0.001
Smoker	949 (28.49%)	352 (37.09%)	597 (62.91%)		
Non-smoker	2382 (71.51%)	1135 (47.65%)	1247 (52.35%)		
Drinking status				31.64	<0.001
Drinker	1006 (30.20%)	375 (37.28%)	631 (62.72%)		
Non-drinker	2325 (69.80%)	1112 (47.83%)	1213 (52.17%)		
Present occupation				47.30	<0.001
No	1711 (51.37%)	862 (50.38%)	849 (49.62%)		
Farming	1380 (41.42%)	537 (38.91%)	843 (61.09%)		
Non-farming occupations	240 (7.21%)	88 (36.67%)	152 (63.33%)		
Medical insurance type				1.60	0.451
UEBMI	87 (2.61%)	38 (43.68%)	49 (56.32%)		
RBMI	3189 (95.74%)	1429 (44.81%)	1760 (55.19%)		
Others	55 (1.65%)	20 (36.36%)	35 (63.64%)		
Online frequency				1.20	0.550
Never	2528 (75.89%)	316 (42.88%)	421 (57.12%)		
1~4 times per month	66 (1.98%)	30 (45.45%)	36 (54.55%)		
Every day	737 (22.13%)	1141 (45.13%)	1387 (54.87%)		
Obesity status				85.71	<0.001
Underweight	110 (3.30%)	33 (30.00%)	77 (70.00%)		
Normal	1309 (39.30%)	485 (37.05%)	824 (62.95%)		
Overweight	1356 (40.71%)	646 (47.64%)	710 (52.36%)		
Obesity	556 (16.69%)	323 (58.09%)	233 (41.91%)		
Amount of exercise				0.85	0.652
Little	2491 (74.78%)	1121 (45.00%)	1370 (55.00%)		
Middle	804 (24.14%)	352 (43.78%)	452 (56.22%)		
Big	36 (1.08%)	14 (38.89%)	22 (61.11%)		

Note: Q1, <5000; Q2, 5000~10,000; Q3, ≥10,000; UEBMI, Basic Medical Insurance for Urban Employee; RBMI, Basic Medical Insurance for Urban and Rural Residents; NO-CMM, No cardiometabolic multimorbidity.

**Table 2:** Fitting index and class probability of five category models for latent profile analysis of dietary patterns.

Class	Loglikelihood	AIC	BIC	aBIC	Entropy	LMRT (*p*)	BLRT (*p*)	Class Probability (%)
1	−56,711.82	113,471.64	113,618.31	113,542.05	—	—	—	1.00
2	−55,772.74	111,619.48	111,845.59	111,728.02	0.61	<0.001	<0.001	0.49/0.51
3	−54,357.39	108,814.78	109,120.33	108,961.46	0.90	<0.001	<0.001	0.23/0.59/0.18
4	−54,008.12	108,142.24	108,527.24	108,327.06	0.88	<0.001	<0.001	0.17/0.23/0.53/0.07
5	−53,628.85	107,409.71	107,874.15	107,632.66	0.88	0.007	0.007	0.20/0.02/0.62/0.07/0.09

**Table 3:** Average attribution probability (average posterior probability) of the most likely category members (rows) by latent category (column).

Class	*n*	(%)	Attribution Probability			
			Class 1	Class 2	Class 3	Class 4
Class 1	571	17.14	0.91	0.00	0.08	0.00
Class 2	754	22.64	0.00	0.99	0.01	0.00
Class 3	1770	53.14	0.04	0.01	0.92	0.03
Class 4	236	7.08	0.01	0.02	0.10	0.88

Note: Class 1, Low Consumption of All Foods Dietary Pattern; Class 2, High Dairy, Egg, and Red Meat Consumption, Low Vegetable and High-Salt Consumption Dietary Pattern; Class 3, High Egg, Vegetable, and Grain Consumption, Low Dairy and White Meat Consumption Dietary Pattern; Class 4, High Meat and Fish Consumption, Low Dairy and High-Salt Consumption Dietary Pattern.

**Table 4:** Prevalence of CMM (%) by dietary patterns among rural older adults.

Variable	CMM	NO-CMM	*χ*2	*p*
Class 1	276 (48.34%)	295 (51.66%)	8.37	0.03
Class 2	353 (46.82%)	401 (53.18%)		
Class 3	764 (43.16%)	1006 (56.84%)		
Class 4	94 (39.83%)	142 (60.17%)		

Note: Class 1, Low Consumption of All Foods Dietary Pattern; Class 2, High Dairy, Egg, and Red Meat Consumption, Low Vegetable and High-Salt Consumption Dietary Pattern; Class 3, High Egg, Vegetable, and Grain Consumption, Low Dairy and White Meat Consumption Dietary Pattern; Class 4, High Meat and Fish Consumption, Low Dairy and High-Salt Consumption Dietary Pattern. NO-CMM, No cardiometabolic multimorbidity.

**Table 5:** Multivariate logistic regression of dietary patterns associated with the prevalence of CMM among rural older adults.

Heading	Crude Model 1		Adjusted Model 2	
	OR (95%CI)	*p*	OR (95%CI)	*p*
Dietary patterns				
Class 1	Ref.		Ref.	
Class 2	0.94 (0.76–1.17)	0.583	0.87 (0.69–1.10)	0.252
Class 3	0.81 (0.67–0.98)	0.031	0.80 (0.66–0.98)	0.028
Class 4	0.71 (0.52–0.96)	0.028	0.70 (0.51–0.97)	0.034

Note: OR denotes to odds ratio; CI denotes to confidence interval; Crude Model 1-non adjusted model; Adjusted Model 2—model adjusted for age, sex, highest education, marital status, live arrangement, personal income, smoking status, drinking status, present occupation, medical insurance type, online frequency, obesity status, amount of exercise. Class 1, Low Consumption of All Foods Dietary Pattern; Class 2, High Dairy, Egg, and Red Meat Consumption, Low Vegetable and High-Salt Consumption Dietary Pattern; Class 3, High Egg, Vegetable, and Grain Consumption, Low Dairy and White Meat Consumption Dietary Pattern; Class 4, High Meat and Fish Consumption, Low Dairy and High-Salt Consumption Dietary Pattern.

## Data Availability

The data of this study are available to researchers upon reasonable request to corresponding authors.
